# Modulation of chemokine gradients by apheresis redirects leukocyte trafficking to different compartments during sepsis, studies in a rat model

**DOI:** 10.1186/cc13969

**Published:** 2014-07-03

**Authors:** Zhi-Yong Peng, Jeffery V Bishop, Xiao-Yan Wen, Michele M Elder, Feihu Zhou, Anan Chuasuwan, Melinda J Carter, Jason E Devlin, A Murat Kaynar, Kai Singbartl, Francis Pike, Robert S Parker, Gilles Clermont, William J Federspiel, John A Kellum

**Affiliations:** 1 The Center of Critical Care Nephrology, Department of Critical Care Medicine, University of Pittsburgh School of Medicine, Pittsburgh, Pennsylvania, USA; 2 The CRISMA (Clinical Research, Investigation, and Systems Modeling of Acute Illness) Center, Department of Critical Care Medicine, University of Pittsburgh School of Medicine, Pittsburgh, Pennsylvania, USA; 3 Center for Biological Imaging, Department of Cell Biology and Physiology, University of Pittsburgh School of Medicine, Pittsburgh, Pennsylvania, USA; 4 McGowan Institute for Regenerative Medicine, University of Pittsburgh School of Medicine, Pittsburgh, Pennsylvania, USA; 5 Department of Chemical and Petroleum Engineering, University of Pittsburgh Swanson School of Engineering, Pittsburgh, Pennsylvania, USA; 6 Department of Bioengineering, University of Pittsburgh Swanson School of Engineering, Pittsburgh, Pennsylvania, USA; 7 Clinical and Translational Science Institute, University of Pittsburgh, 604 Scaife Hall, 3550 Terrace Street, Pittsburgh, PA 15261, USA

## Abstract

**Introduction:**

Prior work suggests that leukocyte trafficking is determined by local chemokine gradients between the nidus of infection and the plasma. We recently demonstrated that therapeutic apheresis can alter immune mediator concentrations in the plasma, protect against organ injury, and improve survival. Here we aimed to determine whether the removal of chemokines from the plasma by apheresis in experimental peritonitis changes chemokine gradients and subsequently enhances leukocyte localization into the infected compartment, and away from healthy tissues.

**Methods:**

In total, 76 male adult Sprague–Dawley rats weighing 400 g to 600 g were included in this study. Eighteen hours after inducing sepsis by cecal ligation and puncture, we randomized these rats to apheresis or sham treatment for 4 hours. Cytokines, chemokines, and leukocyte counts from blood, peritoneal cavity, and lung were measured. In a separate experiment, we labeled neutrophils from septic donor animals and injected them into either apheresis or sham-treated animals. All numeric data with normal distributions were compared with one-way analysis of variance, and numeric data not normally distributed were compared with the Mann–Whitney *U* test.

**Results:**

Apheresis significantly removed plasma cytokines and chemokines, increased peritoneal fluid-to-blood chemokine (C-X-C motif ligand 1, ligand 2, and C-C motif ligand 2) ratios, and decreased bronchoalveolar lavage fluid-to-blood chemokine ratios, resulting in enhanced leukocyte recruitment into the peritoneal cavity and improved bacterial clearance, but decreased recruitment into the lung. Apheresis also reduced myeloperoxidase activity and histologic injury in the lung, liver, and kidney. These Labeled donor neutrophils exhibited decreased localization in the lung when infused into apheresis-treated animals.

**Conclusions:**

Our results support the concept of chemokine gradient control of leukocyte trafficking and demonstrate the efficacy of apheresis to target this mechanism and reduce leukocyte infiltration into the lung.

## Introduction

Recruitment of leukocytes to the location of pathogens is a critical step in the innate immune response to infection [1]. Neutrophils and monocytes in particular are actors in the complex inflammatory network that typifies sepsis [1-4]. However, leukocyte trafficking during sepsis is altered [3], and these cells (especially neutrophils and monocytes) are often seen as both helpful and harmful in human sepsis [2,4,5]. When the response to infection is contained and controlled, pathogens are rapidly cleared, and healthy tissues are largely spared. Conversely, widespread activation of neutrophils and monocytes leads to multiple organ injury and failure [5-7]. Directing leukocyte recruitment to the site of infection and away from healthy tissue can therefore be seen as a fundamental strategy to treat sepsis.

Neutrophils and monocytes find their way to the site of infection largely by sensing various chemical attractants. Perhaps the most important are the chemokines classified as chemokine (C-X-C motif) ligand 1 (CXCL1) [8], ligand 2 (CXCL2) [9], and ligand 8 (CXCL8) [10] for neutrophils and chemokine (C-C motif) ligand 2 (CCL2) for monocytes [11]. Numerous studies have suggested that leukocyte recruitment is dramatically influenced by the relative proportions of these molecules between tissue and plasma [12-16]. Within a physiologic range, as the gradient between infected tissue and plasma increases, leukocyte recruitment is enhanced, and pathogen clearance is improved. Conversely, as the gradient between noninfected tissue and plasma increases, inflammation and injury to these tissues are worsened.

Recently, Craciun and colleagues [17] demonstrated that local injection of CXCL1 and CXCL2 into the peritoneum after cecal ligation and puncture (CLP) resulted in enhanced neutrophil localization, decreased bacterial growth, and improved survival in mice. However, translation of these findings into clinical practice would be challenging. Alternatively, it is possible to change the chemokine gradients by reducing the plasma concentrations by using apheresis. In a recent meta-analysis, blood purification, especially hemoperfusion, significantly improved the survival in patients with sepsis [18]. CytoSorbbeads (CytoSorbents, Monmouth Junction, NJ, USA), are composed of a novel highly adsorptive and biocompatible polymer and have the potential to remove the multiple inflammatory mediators from the bloodstream. Our prior studies have optimized the adsorptive efficacy of CytoSorb beads and have demonstrated that therapeutic apheresis by using a hemoadsorption (HA) device composed of these beads could alter immune-mediator concentrations in the plasma, protect against organ injury, and improve survival [19-22]. However, acute removal of common sepsis mediators did not explain the entire effect of this treatment, and other mechanisms must exist [21].

Thus, we sought to alter chemokine gradients by reducing the plasma concentrations by using an apheresis system designed with CytoSorb beads in a CLP model in the rat and to determine whether this mechanism would result in improved leukocyte trafficking and bacterial clearance.

## Material and methods

### Experimental design and study overview

**Figure 1:**
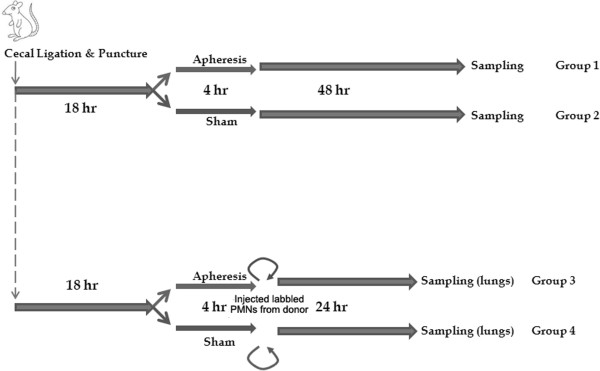
**Individual experimental groups.** This study is composed of two experimental phases. In the first experiment, 18 hours after CLP, animals (groups 1 and 2) were randomly assigned to receive either apheresis by hemoadsorption (HA) or sham treatment for 4 hours. Animals were then killed for sampling 48 hours after treatment. In the second experiment, 18 hours after CLP, animals were randomly assigned to receive either apheresis by HA or sham treatment for 4 hours. Labeled neutrophils from a separate group of septic donor animals were injected into all the treated animals. At 24 hours after treatment, animals were then killed for measurement of labeled neutrophils in lung tissue.

Our study involved two experimental phases. In the first experiment, we randomized animals (Groups 1 and 2) to receive either HA or sham treatment for 4 hours beginning 18 hours after CLP. We then killed animals at a fixed time point 48 hours after treatment to collect fluid from the abdomen and lungs and to collect tissue. Then in the second experiment, we again randomized animals (Groups 3 and 4) to receive either HA or sham treatment for 4 hours beginning 18 hours after CLP. However, in this experiment, we obtain neutrophils from a separate group of septic donor animals, labeled and injected them to all animals immediately *after* treatment. Finally, 24 hours later, we sacrificed animals for measurement of labeled neutrophils in lung tissue. A detailed experimental protocol is shown in Figure 1.

### Animal care and surgical procedures

All animal experiments were conducted according to an Institutional Animal Care and Use Committee of the University of Pittsburgh (protocol 1009994) and in adherence to the National Institutes of Health Guide for the Care and Use of Laboratory Animals.

We anesthetized 76 adult (24 to 28 weeks old weight, 450 to 550 g), male, Sprague–Dawley rats with pentobarbital sodium (50 mg/kg intraperitoneally). We performed a standard CLP procedure, as described previously [21], whereby 25% of the cecum was ligated, and two punctures were performed by using a 20-gauge needle. The abdomen was then closed, and 10 ml/kg saline was given subcutaneously as fluid resuscitation. Topical anesthetic (lidocaine/prilocaine) was applied to the surgical wound once immediately after the surgery to reduce the postoperative pain. Rats were returned to their cages and allowed food and water *ad libitum*. Eighteen hours after CLP, animals were reanesthetized with pentobarbital sodium. The femoral vein and the internal jugular vein were isolated by dissection and cannulated with 1.27-mm polyethylene-90 tubing for use of extracorporeal circulation.

### Experimental protocol

Eighteen hours after CLP, animals were randomly assigned to receive either apheresis by hemoadsorption (HA) or sham treatment for 4 hours. The extracorporeal circulation was driven by a mini-pump (Fisher Scientific, Pittsburgh, PA, USA) from internal jugular vein to femoral vein at a blood-flow rate of 0.8 to 1.0 ml/min (scaled to approximately 140 ml/min in a 70-kg human). In the HA groups, the extracorporeal circulation passed through a 1-ml cartridge containing CytoSorb polymer beads (CytoSorbents, Monmouth Junction, NJ, USA). In the sham group, the blood was passed through tubing with the same dead space as the cartridge column. The extracorporeal circuit was primed with saline containing 20 IU heparin/ml (total volume about 2 ml). After 4 hours of therapy (or sham), the treatment was stopped, and the rats were returned to the animal facility, and given access to food and water.

Forty animals (*n* = 20 per group, groups 1 and 2) were reanesthetized 48 hours after treatment and underwent additional procedures as follows: First, blood was taken for bacterial culture and chemokine measurements. Next, 30 ml of cooled (4°C) 2.6 m*M* ethylenediaminetetraacetic acid (EDTA)-phosphate-buffered saline solution (PBS) (0.02 *M*, pH 7.2) was injected into the abdominal cavity, and the abdomen was massaged for 10 minutes. The peritoneal lavage fluid was recovered by peritoneal puncture. The lavage fluid was then used for bacterial culture and cytokine/chemokine measurements.

Next, 15 ml of cooled 2.6 m*M* EDTA-PBS was injected into the trachea for bronchoalveolar lavage (BAL). The aspirated peritoneal fluid and BAL fluid were then centrifuged at 500 *g* for 5 minutes. The sediments were reserved for flow-cytometry analysis, and supernatants were collected and kept for assay of cytokines/chemokines.

Finally, animals were killed, and lungs, liver, and kidneys were collected.

Thirty-six animals were subjected to the same CLP procedure. Neutrophils from septic donors (one donor for two different treated animals each time) were isolated, labeled, and then injected into animals treated with apheresis or sham (groups 3 and 4; *n* = 12 each) immediately after completing a course of therapy. These treated animals were then killed, and lungs were harvested the next day. Neutrophils in the lungs were observed by using immunofluorescence microscopy.

### Chemokine/cytokine measurements and bacterial load in compartments

Chemokines (CXCL1, CXCL2, and CCL2), and cytokines, including interleukin-1 beta (IL-1β), interleukin-6 (IL-6), and tumor necrosis factor alpha (TNF-α), from plasma, peritoneal fluid (PF), and BAL fluid were measured with multiplex bead-based Luminex assays (Invitrogen, Camarillo, and Affymetrix, Fremont, CA, USA). Assays were performed by using a Bio-Rad Bio-Plex 200 instrument and analyzed with Bio-Plex Manager 4.0 software (Hercules, CA, USA). Blood and peritoneal lavage fluid was serially diluted 1:10 and plated on Tryptic Soy Agar (TSA) plates. Plates were incubated for 48 hours at 37°C and 5% CO_2_. Colony-forming units (CFUs) were enumerated on plates containing 30 to 300 colonies, and counts were adjusted to original concentration.

### Complete blood counts

Complete blood counts (white blood cells, red blood cells, hemoglobin, hematocrit, and platelets) from central veins were measured with an Abbott Automatic Analyzer (Abbott Laboratory, Abbott Park, IL, USA).

### Leukocyte counts with differential and leukocyte-function tests

Leukocyte counts were performed on BAL and peritoneal lavage fluid by using a hemocytometer. Differential counts were performed with flow cytometry. Cells were washed and centrifuged for 3 cycles and then stained with PE-Cy™5 mouse anti-rat CD45 (OX-1), PE mouse anti-rat granulocyte (RP-1), and Biotin mouse anti-rat mononuclear followed by FITC streptavidin staining (all from BD Pharmigen, San Diego, CA, USA). Cells were gated on CD45. CD45 cells were analyzed for positive expression of either the granulocyte or mononuclear markers.

Leukocyte functions, including chemotaxis, phagocytosis, and respiratory burst, were measured. The respiratory burst is the rapid release of reactive oxygen species (ROS) from leukocytes and is evaluated with the release of ROS. Neutrophils were isolated by using density gradients and resuspended in Hank Balanced Salt Solution (HBSS). For chemotaxis tests, a 1.5% agarose gel was created, and three wells placed in a single line in the gel. Then 10^5^ cells were added to the central well, and 10 μL of 1 pmol formyl-methionyl-leucyl-phenylalanine (FMLP) or HBSS was added to the outer wells and incubated. Cells moving toward each well were counted, and results expressed as a ratio of cells moving toward the FMLP well compared with the control well. For the phagocytosis test, 10^7^ cells were incubated with 10^8^ fluorescein isothiocyanate-labeled *Pseudomonas aeruginosa* (Sigma-Aldrich Co., St. Louis, MO, USA) for 30 minutes. Cells were then fixed and measured with flow-cytometry. For ROS, 10^7^ cells were incubated with dihydroethidium for 30 minutes, before being fixed and analyzed. Flow-cytometry samples were gated for neutrophil size, and the mean fluorescent intensity of the cells was measured.

### Determination of neutrophil infiltration in tissues

Organs removed from groups 1 and 2 were weighed and homogenized in lysis buffer. Cell lysates were sonicated and centrifuged. The separated supernatant and pellet were kept at −80°C until used. The protein concentrations from supernatant and pellet were determined by using bicinchoninic acid kit (Pierce, Rockford, IL, USA) according to the manufacturer’s instruction. Myeloperoxidase (MPO) activity was measured from the pellets. Neutrophil infiltration was assessed with MPO activity by using a spectrophotometric method [23]. The prepared tissue samples were mixed with phosphate buffer containing *o*-dianisidine dihydrochloride and hydrogen peroxidase. One unit of peroxidase activity was defined as the amount of enzyme decomposing 1 μmol hydrogen peroxidase per minute at 25°C. Decomposition of hydrogen peroxide was calculated from the oxidation of *o*-dianisidine by using an absorption coefficient of 11.3 L/mol per cm at 460 nm. MPO was presented as U/ml/mg tissue protein.

### *In vivo* labeling of leukocytes and evaluation of the trafficking of these labeled leukocytes

Neutrophils were isolated by using density gradients. Neutrophil purity was >85%. Isolated neutrophils were stained with Cell Tracker Red [24] (Invitrogen) according to the manufacturer’s instructions. Labeled cells were injected into animals immediately after apheresis or sham treatment. Twenty-four hours after injection of labeled neutrophils, animals were killed, and lungs inflated and fixed in 2% PFA for 4 hours at 4°C. After fixation, lungs were transferred to 30% sucrose solution overnight at 4°C. The samples were then frozen by using liquid nitrogen along with 2-methylbutane and stored at −80°C. The lungs were cryosectioned on a cryostat, Micron model HM550 at 7 microns thickness. Next the slides were stained with 647 Cy5 phalloidin markers to label the actin and Hoechst nuclear stain to label the nucleus. Slides were coverslipped using Gelvatol and stored at 4°C to dry overnight.

Ten sections were placed onto slides and stained with 4',6-diamidino-2-phenylindole (DAPI). The sections were chosen at random during the staining process. Slides were evaluated using 10 field views per slide totaling 100 field views per animal. Field views were selected using a zig-zag pattern through the tissue. Areas of large spaces were avoided and fields were excluded if they crossed an exterior border of the lung. Pictures were taken using an Olympus upright epi fluorescence microscope (model BX51, Olympus Imaging America, Center Valley, PA) and analyzed by a researcher (J-E, D) blinded to the animal treatment state using software of MagnaFire version 2.1 (Optronics, Goleta, CA).

### Histology

Liver and kidney sections were fixed in 10% neutral buffered formalin, dehydrated in graded anhydrous absolute ethanol, and embedded in paraffin. Lungs were inflated and fixed with Zinc Fixative (BD Pharmingen, San Diego, CA). Histological sections (5 μm) were stained with hematoxylin, eosin and periodic acid Schiff. The histology was assessed by a pathologist (X-Y, W) blinded to the intervention.

### Ex-vivo bacterial adsorption

Escherichia coli (E. coli, Sigma-Aldrich Co., St Louis, MO) were reconstituted with Bacto Tryptic Soy Broth (TSB) and then incubated overnight. Optical density was checked every 15 minutes until desired E. coli concentration was achieved. The E. coli suspension was diluted 1:25000, and 7 mL of the dilution added to a 15 mL conical tube. A sterilized closed circuit containing the column with the CytoSorb polymer beads (HA) or empty column (sham) through the conical tube (reservoir) was set up. The flow rate was controlled at 0.8 ml/min with the mini-pump. 50 uL of duplicate samples from pre-column, post-column and reservoir were collected before circulation, and 2 and 4 hours after circulation began. These samples were put on agar plates and allowed to incubate at 37°C. The CFUs were counted the following morning.

### Statistical analyses

Numeric data not normally distributed were expressed as median and range, natural log transformed, and compared with Mann–Whitney *U* test. All numeric data with normal distribution (including cytokine and chemokine data after natural log transformation) were expressed as mean ± standard error (SE), and compared with one-way analysis of variance and the *post hoc* Student-Neuman-Keuls test. All analyses were performed by using SPSS statistical software package (SPSS for Windows; Chicago, IL, USA). *P* values below 0.05 were considered to be statistically significant.

## Results

### Apheresis changed the cytokine and chemokine levels in different compartments and altered the ratios of local to systemic chemokine concentrations

**Figure 2:**
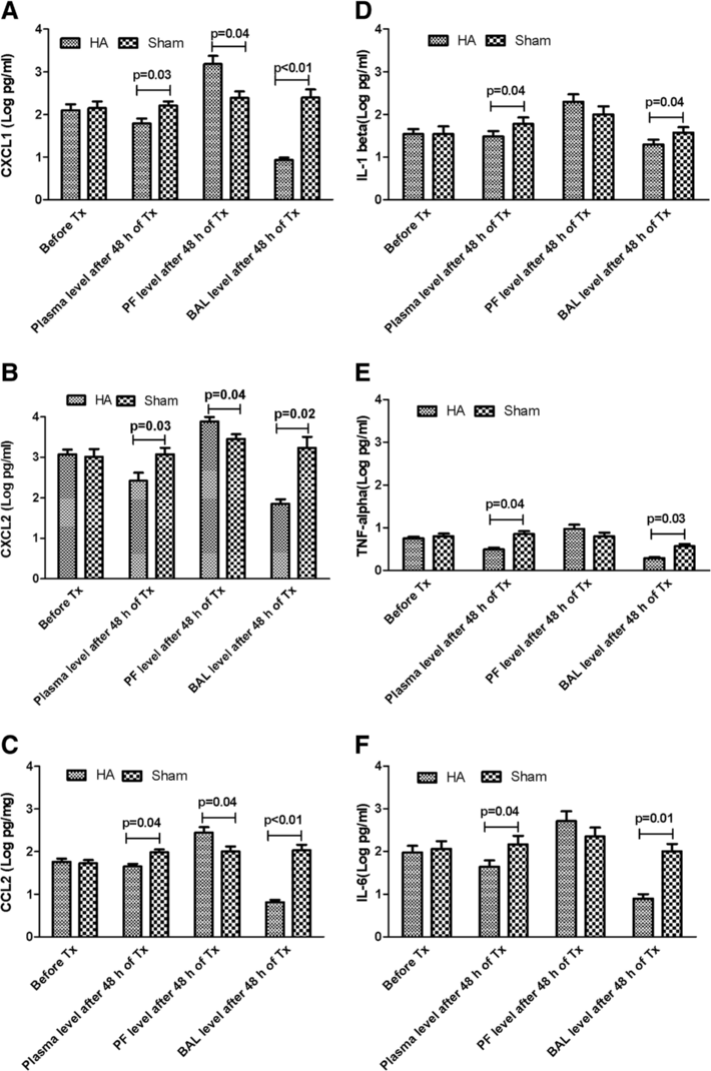
**Effects of apheresis on cytokines and chemokines in different compartments (data are expressed as mean ± SE after being natural log transformed, *n*** **= 14 to 18 each).** Eighteen hours after CLP, animals were randomly assigned to receive either apheresis by hemoadsorption (HA) or sham treatment for 4 hours. Animals were then killed for sampling 48 hours after treatment. Shown are the comparisons among blood, peritoneal fluid (PF), and bronchoalveolar lavage fluid (BAL) after treatments. **(A)** CXCL1; **(B)** CXCL2; **(C)** CCL2; **(D)** IL-1β; **(E)** TNF-α; and **(F)** IL-6. Tx, treatment. *“*Before Tx” is the time point when samples were obtained at 18 hours after CLP but before initiation of apheresis.

**Figure 3:**
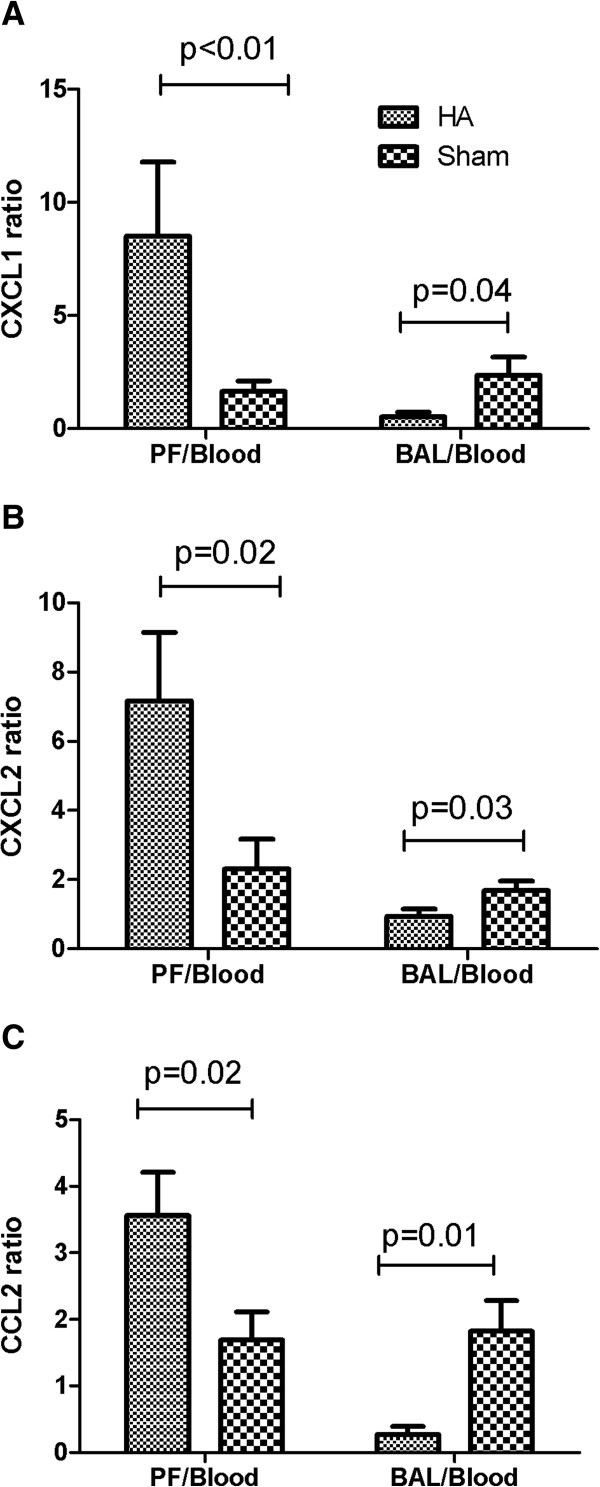
**Effects of apheresis on the ratios of local to systemic chemokine concentrations (mean ± SE, *n*** **= 14 to 18 each).** Eighteen hours after CLP, animals were randomly assigned to receive either apheresis by hemoadsorption (HA) or sham treatment for 4 hours. Animals were then killed for sampling 48 hours after treatment. Shown are peritoneal fluid (PF)-to-blood and bronchoalveolar lavage fluid (BAL)-to-blood ratios for **(A)** CXCL1; **(B)** CXCL2; and **(C)** CCL2.

Cytokines (IL-1β, TNF-α, and IL-6) and chemokines (CXCL1, CXCL2, and CCL2) in plasma and lung were significantly decreased with apheresis (Figure 2; *P* < 0.05). Apheresis also increased chemokines (CXCL1, CXCL2, and CCL2) in the peritoneal cavity (Figure 2; *P* < 0.05). Compared with sham, apheresis treatment significantly increased the ratios of chemokines from peritoneal fluid to circulating plasma: CXCL1 (8.51 versus 1.65; *P* < 0.01, Figure 3A), CXCL2 (7.17 versus 2.32; *P* = 0.02, Figure 3B) and CCL2 (3.56 versus 1.69; *P* = 0.02; Figure 3C). It is important that, compared with sham, apheresis also significantly decreased the ratios of BAL to plasma chemokines: CXCL1 (0.52 versus 2.37; *P* = 0.04; Figure 3A), CXCL2 (0.94 versus 1.69, *P* = 0.03; Figure 3B) and CCL2 (0.27 versus. 1.82; *P* = 0.01; Figure 3C).

### Apheresis redirected leukocyte trafficking into different compartments and improved bacterial clearance from the peritoneal cavity

**Figure 4:**
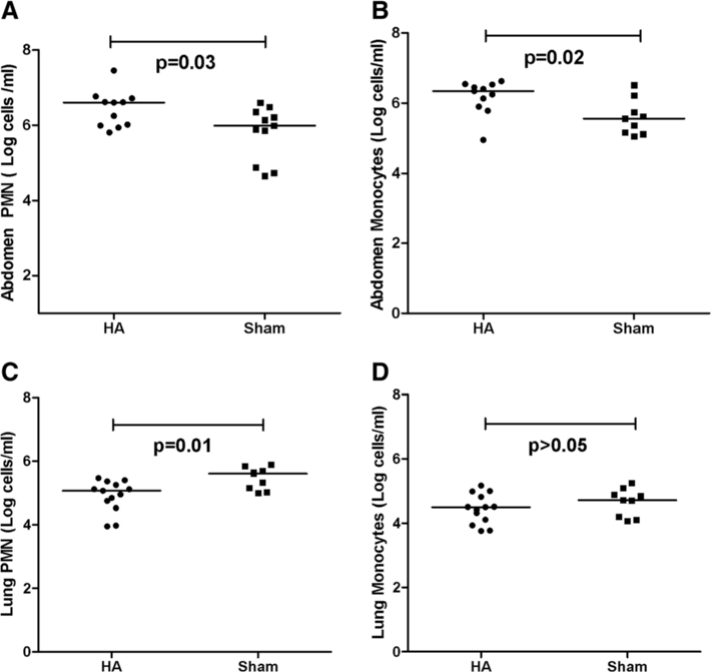
**Effects of apheresis on leukocyte influx into the peritoneal cavity and lung.** Eighteen hours after CLP, animals were randomly assigned to receive either apheresis by HA or sham treatment for 4 hours. Animals were then killed for sampling 48 hours after treatment. Cells were washed and centrifuged for three cycles and then stained with PE-Cy5 mouse anti-rat CD45 (OX-1), PE mouse anti-rat granulocyte (RP-1) and Biotin mouse anti-rat mononuclear followed by FITC streptavidin staining. Cells were gated on CD45. CD45 cells were analyzed for positive expression of either the granulocyte or mononuclear markers. Shown are **(A)** neutrophils; and **(B)** monocytes in the peritoneal cavity; **(C)** neutrophils and **(D)** monocytes in the lung. Comparisons are between apheresis (HA) and sham. Medians and ranges of cells per milliliter (natural log transformed, *n* = 14 to 18 each) are shown.

To determine whether modulation of chemokine gradients resulted in changes in leukocyte trafficking, we then measured the number of leukocytes in the peritoneum and lungs. Figure 4 shows that peritoneal fluid from apheresis-treated animals contained more neutrophils as compared with that from sham-treated animals (4.0 × 10^6^/ml versus 0.97 × 10^6^/ml; *P* = 0.03; Figure 4A) and more monocytes (2.2 × 10^6^/ml versus 0.36 × 10^6^/ml; *P* = 0.02, Figure 4B). In contrast to the changes in the peritoneal cavity, fewer neutrophils (1.16 × 10^5^/ml apheresis versus 4.0 × 10^5^/ml sham; P = 0.01; Figure 4C) were in the lungs after apheresis treatment. However, no significant difference was found in monocytes in the lungs between the apheresis and sham-treated animals (3.1 × 10^3^/ml versus 5.1 × 10^3^/ml; *P* = 0.35; Figure 4D).

**Figure 5:**
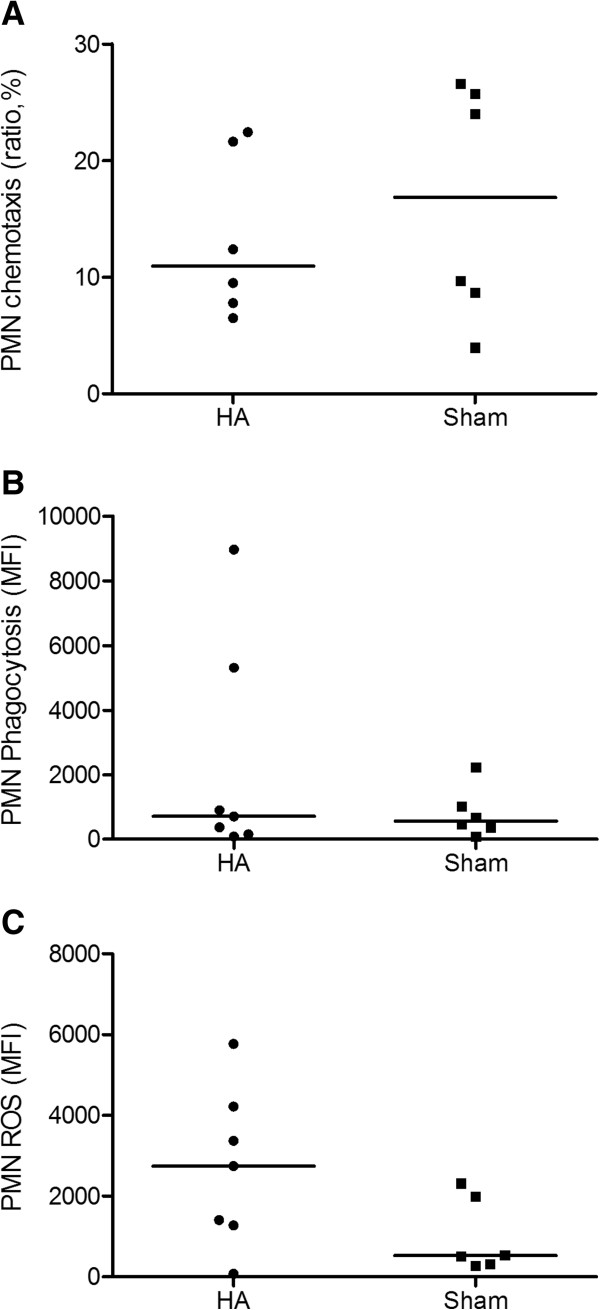
**Effects of apheresis on PMN function (median and ranges, *n*** **= 7).** Eighteen hours after CLP, animals were randomly assigned to receive either apheresis by HA or sham treatment for 4 hours. Animals were then killed for sampling 48 hours after treatment. **(A)** PMN chemotaxis expressed as ratio (percentage) of cells migrating toward chemoattractant to cells migrating away from chemoattractant. **(B)** PMN phagocytosis expressed as mean fluorescent intensity (MFI). **(C)** PMN oxidative burst (ROS) expressed as mean fluorescent intensity (MFI).

**Figure 6:**
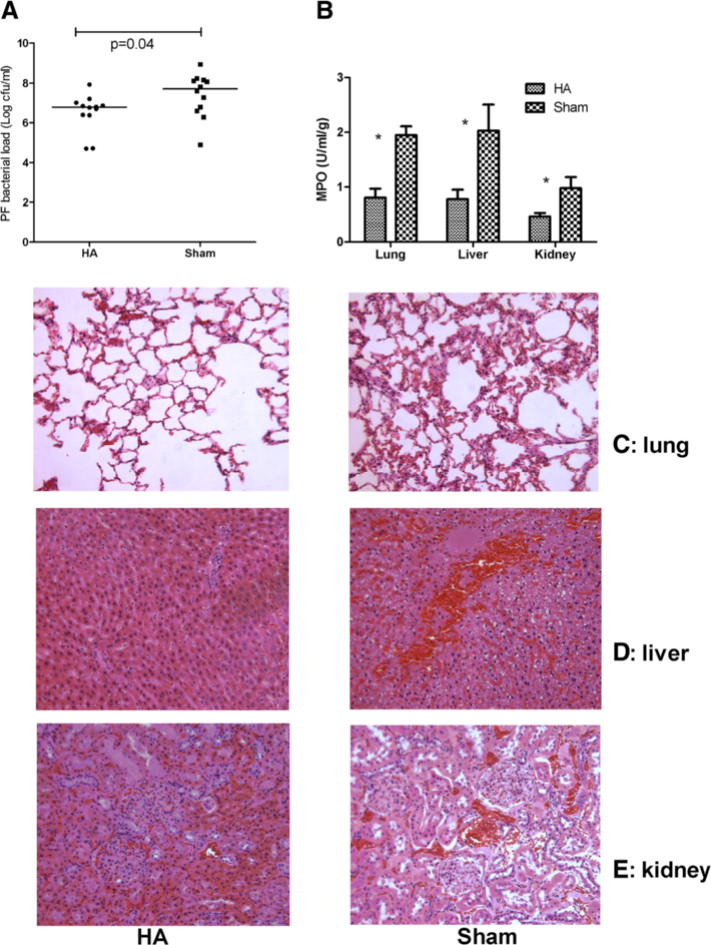
**Effects of apheresis on the bacterial clearance, neutrophil infiltration, and histopathology.** Eighteen hours after CLP, animals were randomly assigned to receive either apheresis (HA) or sham treatment for 4 hours. Animals were then killed for sampling 48 hours after treatment. **(A)** Bacterial load in the peritoneal fluid (PF) (medians and ranges for colony-forming units per milliliter (natural log transformed, *n* = 12 to 16 each). **(B)** Myeloperoxidase (MPO) activity (mean ± SE, U/ml/mg; *n* = 20 each). *P < 0.05, apheresis (HA) versus sham in lung, liver, and kidney. **(C)** Lung histology (*n* = 4 to 6 each). Sham showed neutrophil infiltration and hemorrhage not seen with apheresis (HA). **(D)** Liver histology (*n* = 4 to 6 each). Apheresis (HA) showed milder swelling of hepatocytes and necrosis compared with sham. **(E)** Kidney histology (*n* = 4 to 6 each). Apheresis (HA) resulted in less vacuolization in tubules compared with sham.

To investigate whether apheresis directly altered neutrophil function, we measured chemotaxis, phagocytosis, and oxidative burst. No differences were found in PMN oxidative burst or in other measures in neutrophils taken from animals treated with apheresis versus sham (Figure 5). Apheresis also resulted in less bacterial growth from the peritoneal fluid (6.05 × 10^6^cfu/ml versus 52.15 × 10^6^cfu/ml; *P* = 0.04; Figure 6A). Apheresis did not alter white blood cell or platelet counts (Table 1). In addition, no bleeding-related adverse events were found.

**Table 1:** **Effects of apheresis on complete blood cells (*n*** **= 6, mean and SE)**

	**Before apheresis**		**After 4-hour apheresis**		***P *** **values**	
	**HA**	**Sham**	**HA**	**Sham**		
RBC (×10^12^/L)	3.92 ± 0.18	3.75 ± 0.36	3.88 ± 0.17	3.83 ± 0.31	0.88^1^	0.89^2^
WBC (×10^9^/L)	8.38 ± 0.70	8.17 ± 0.48	7.17 ± 0.57	8.73 ± 0.53	0.21^1^	0.09^2^
Platelets (×10^9^/L)	371 ± 39	341 ± 29	290 ± 32	336 ± 32	0.11^1^	0.33^2^

HA, apheresis by hemoadsorption; Sham, sham treatment; RBC, red blood cell; WBC, white blood cell.

^1^: before values versus after values in HA group; ^2^: HA versus Sham after treatments.

### Apheresis did not adsorb the bacteria directly

To explore where the improved bacterial clearance by the apheresis is to the result of the increased leukocyte trafficking or direct adsorption, we ran an *ex vivo* bacterial adsorption experiment. After 2 and 4 hours, no significant differences were found in bacterial loads between the pre- and postcolumn with beads (60.58 × 10^6^ versus 61.38 × 10^6^ at 2 hours and 224.40 × 10^6^ versus 222.00 × 10^6^ at 4 hours; *P* > 0.05; Table 2). Moreover, the bacterial loads in the reservoirs between the sham and apheresis at 2 hours (51.20 × 10^6^ versus 54.75 × 10^6^; *P* = 0.97) and 4 hours of circulation (241.70 × 10^6^ versus 218.20 × 10^6^; *P* = 0.56) were not different (Table 3).

**Table 2:** **Effects of apheresis on *ex vivo *bacterial removal (*n*** **= 6, mean and SE); Changes in bacterial load between pre- and post-columns after 2- and 4-hour apheresis**

	**After 2-hour apheresis (×10**^**6**^ **CFU/ml)**		**After 4-hour apheresis (×10**^**6**^ **CFU/ml)**		***P *** **values**^**1**^
	**Pre-columns**	**Post-columns**	**Pre-columns**	**Post-columns**	
Sham	79.68 ± 13.88	73.43 ± 14.52	267.60 ± 35.63	260.70 ± 38.06	0.76; 0.90
HA	60.58 ± 4.27	61.38 ± 6.21	222.40 ± 11.60	222.00 ± 20.65	0.92; 0.99
*P* values^2^	0.22	0.46	0.26	0.39	

HA, apheresis by hemoadsorption; Sham, sham treatment.

^1^: Precolumn values versus postcolumn values at the same time; ^2^HA versus Sham.

**Table 3:** **Effects of apheresis on *ex vivo *bacterial removal (*n*** **= 6, mean and SE); Changes in bacterial loads in the reservoir after 2- and 4-hour apheresis**

	**Before apheresis (CFU/ml)**	**After 2-our apheresis (CFU/ml)**	**After 4-hour apheresis (CFU/ml)**
Sham	9.15 × 10^6^ ± 1.68 × 10^6^	51.20 × 10^6^ ± 9.85 × 10^6^	241.70 × 10^6^ ± 35.09 × 10^6^
HA	8.85 × 10^6^ ± 1.31 × 10^6^	50.75 × 10^6^ ± 5.09 × 10^6^	218.20 × 10^6^ ± 17.25 × 10^6^
*P* values	0.89	0.97	0.56

HA, apheresis by hemoadsorption; Sham, sham treatment.

### Apheresis attenuated remote organ neutrophil infiltration and histologic injury

Animals treated with apheresis also exhibited significantly attenuated MPO activity in the lungs (0.80 versus 1.95 U/ml/g; *P* = 0.01, Figure 6B), liver (0.78 versus 2.03 U/ml/g, *P* = 0.02, Figure 6B) and kidney (0.47 versus 0.98 U/ml/g, *P* = 0.04, Figure 6B) compared with the sham-treated animals. Finally, the severity of histopathology (lung, liver, and kidney), although mild overall, was consistent with the MPO results (Figure 6C, D, and E). Sham showed neutrophil infiltration and hemorrhage in lungs, but these changes were not seen with apheresis. Compared with sham treatment, apheresis showed less swelling of hepatocytes and necrosis and resulted in less vacuolization in renal tubules compared with sham.

### Apheresis redirected neutrophils from septic donors away from the lungs

**Figure 7:**
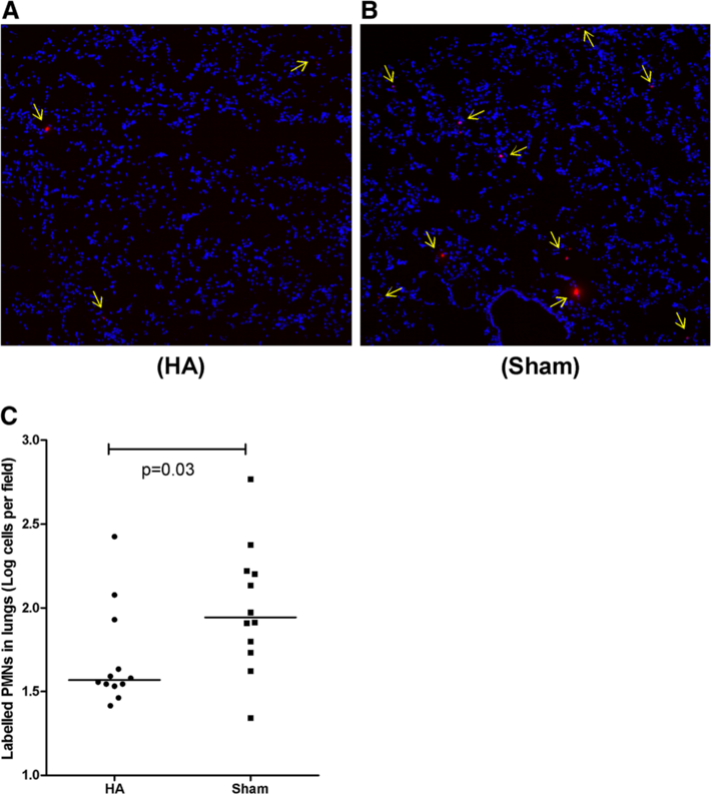
**Effects of apheresis on neutrophil influx into the lungs.** Eighteen hours after CLP, animals were randomly assigned to receive either apheresis (HA) or sham treatment for 4 hours. Labeled neutrophils from septic donor animals were injected after treatment into **(A)** HA and **(B)** sham-treated animals (*n* = 12 each). Neutrophils in the lungs (red color, and arrows) were observed after 24 hours with immunofluorescence microscopy (20× magnifications). **(C)** Comparison of labeled neutrophils infiltrated in the lung between HA and sham-treated septic animals (data expressed as medians and ranges after natural log transformation). Fewer neutrophils were seen in the lungs of HA-treated animals **(A)** compared with sham **(B)**.

Finally, fewer labeled donor neutrophils were seen in lungs taken from apheresis-treated CLP animals compared with sham (37 cells in apheresis versus 88 cells in sham per high-power field, *n* = 12, *P* = 0.03, Figure 7). These results suggest that the decreased neutrophil infiltration into the lungs was due to chemokine gradient differences between groups as a result of apheresis rather than direct effects on the cells.

## Discussion

In this study, we demonstrated that a single 4-hour application of a novel apheresis system removed cytokines and chemokines from plasma, reduced cytokines and chemokines in the lung, and increased chemokines in the peritoneal cavity. Chemokine removal from the plasma compartment by using this technique resulted in increased chemokine gradients between the peritoneal cavity and the plasma; increased leukocyte recruitment into peritoneal space; and decreased bacterial counts in the peritoneal fluid at 48 hours after treatment. Furthermore, we found that chemokine gradients were reversed in the lung (a decreased gradient with active treatment compared with sham), and neutrophil infiltration into the lung was also decreased. Finally, we established that these changes in leukocyte trafficking resulted from changes in the plasma (presumably alterations in chemokine or other mediators) rather than from direct action on the cells themselves. Indeed, donor neutrophils naïve to the intervention exhibited decreased lung infiltration when injected into apheresis-treated animals, although neutrophil function itself was not enhanced by the treatment, as evidenced by lack of change in phagocytosis or bacterial killing.

We used CLP-induced sepsis to test our device in this study because it resembles clinical sepsis [20,21]. The peritonitis that ensues is polymicrobial (for example, *Escherichia coli*, *Proteus mirabilis, Enterococcus* sp.*, Bacteroides fragilis*, and so on) and resembles human disease. We chose CXCL1, CXCL2, and CCL2 as the target chemokines, as these markers represent common chemokines necessary to regulate leukocyte trafficking in rats. These markers are easily detected in this model (CXCL8 not produced by rodents). We started treatment at 18 hours after CLP, as the concentrations of most mediators reach peak levels and the ligated cecum begins to heal after 24 hours [21]. We killed animals for PF and BAL samples at 48 hours after treatments, as our previous study [21] showed that the significant changes in organ function, as well as cytokines/biomarkers, occurred at 48 hours after treatment.

Our results extend the findings of prior studies from our laboratory [19-21] and others [12-16,25,26]. Prior studies found that leukocyte trafficking is highly dependent on cytokine and especially chemokine gradients from the nidus of infection to the circulation [12-16]. Similar to our results, Craciun and colleagues [17] found that increasing these gradients (by direct infusion of chemokines) enhanced neutrophil localization, decreased bacterial growth, and improved survival.

Our prior studies using apheresis as a treatment for sepsis demonstrated improved survival and organ protection [19-21,27] but attempts to understand these effects by using systems modeling revealed that changes in cytokines alone were inadequate to explain the effects on survival [28]. As we scaled back our treatment intensity to a level easily applicable to humans, we found that the effects on circulating cytokines were delayed (by up to 48 hours), even though survival was still improved and appeared to be the result of fewer activated leukocytes in the circulation after therapy [21]. We can now understand these results by observing changes in leukocyte trafficking as a result of apheresis.

Our results are also consistent with an emerging “general model” of sepsis [20,21], which helps explain a number of seemingly conflicting results from clinical and laboratory studies. First, Alves-Filho *et al*. [29] demonstrated that a lethal model of CLP-induced sepsis was associated with less neutrophil recruitment into the peritoneal space compared with a sublethal model, despite less systemic inflammation. Studies in older animals [30,31] also demonstrated that despite greater systemic innate immune system activation, worse bacterial clearance occurred compared with younger animals given the same inoculum. Similarly, in the largest study of its kind to date [32], we found that greater cytokine activity in the plasma (regardless of the cytokine measured) was associated with worse survival in humans with community-acquired pneumonia. The worst survival overall was seen in patients with high levels of both pro- and antiinflammatory cytokines.

Finally, a growing body of evidence indicates the presence of immune-system incompetence in the setting of sepsis despite increased immune system activation [33-36]. What emerges from these diverse findings is a model in which severe local inflammation becomes systemic and leads to uncontrolled immune activation or “spillage” of inflammation to other compartments, thereby disrupting the signal-to-noise relation on which chemokine signaling depends [35,37]. Moreover, once cells are activated in the periphery, they can adhere to similarly activated endothelium and infiltrate into otherwise healthy tissues. Once there, these cells can further stimulate neutrophil recruitment by means of producing chemokines, and perpetuate inflammation and tissue injury [35,37]. Our results show that this process is reversible, leading to reduced organ infiltration and attenuated tissue injury (Figures 6 and 7). We note the impact of computational modeling in pointing out the key role of dysregulated trafficking in sepsis pathophysiology [38], focusing our experimental design on the neutrophil response within a limited time window of intervention. Further insights from modeling pertaining to neutrophil phenotypic evolution during sepsis are the focus of ongoing experimental work.Our investigation has limitations. We elected to conduct these experiments by using a model of sepsis without antibiotics. However, the frequent lack of prehospital antibiotic administration, along with the delays and/or inappropriate use of antibiotics during early hospitalization, make this design relevant. It is also true that sepsis frequently occurs in settings in which organisms are resistant to first-line (or in some cases all) antibiotics, and thus our model has direct relevance to an important clinical problem. We measured only three important chemokines (CXCL1, CXCL2, and CCL2). The roles of other neutrophil chemoattractants such as complement fragments or leukotrienes were not considered in this study. Neither did we evaluate whether bone marrow production of neutrophils was altered by the therapy. Given the small-animal model, we could not perform repeated sampling from the peritoneal cavity and BAL fluid, and could obtain these samples at only one time point (48 hours after treatment) with the death of the animal. We also lack enough evidence to explain how apheresis decreased the chemokine gradients of lung to circulation (blood). It seems more likely that apheresis decreases the levels of pro-inflammatory cytokines (IL-1β, TNF-α, and IL-6; Figure 2D-F), which then results in decreased chemokine production in the lung (Figure 2A-C) and subsequently decreases lung/blood ratio.

Another possibility is that apheresis decreased circulating white blood cells. Our prior *ex vivo* work demonstrated that apheresis adsorbs activated PMNs and monocytes [39]. However, our *in vivo* results do not demonstrate a significant effect on circulating cells (Table 1), and our final experiment used cells naïve to the treatment, taken from untreated donors (Figure 7).

## Conclusion

A novel apheresis device using specific adsorptive biomaterials can effectively restore chemokine gradients toward infected tissue and away from healthy organs in an experimental model of polymicrobial abdominal sepsis. These results should set the stage for future clinical trials.

## Key messages

•Apheresis with specific adsorptive biomaterials can restore chemokine gradients toward infected tissue and away from healthy organs.

•This study supports the concept of chemokine gradient control of leukocyte trafficking and may help explain some of the beneficial effects of this apheresis technology.

•Further studies are needed to explain how apheresis decreases the chemokine gradients of lung to circulation.

## Abbreviations

BAL: Bronchoalveolar lavage; CCL2: C-C motif ligand 2; CFU: colony-forming unit; CLP: cecal ligation and puncture; CXCL1: C-X-C motif ligand 1; CXCL2: C-X-C motif ligand 2; DAPI: 4',6-diamidino-2-phenylindole; EDTA: ethylenediaminetetraacetic acid; FMLP: formyl-methionyl-leucyl-phenylalanine; HA: apheresis with hemoadsorption; HBSS: Hanks balanced salt solution; IL-1β: interleukin-1 beta; IL-6: interleukin-6; MPO: myeloperoxidase; PBS: phosphate-buffered saline solution; PF: peritoneal fluid; ROS: reactive oxygen species; TNF-α: tumor necrosis factor alpha; TSA: tryptic soy agar.

## Competing interests

JAK is a paid consultant for CytoSorbents, and has also received research grants from CytoSorbents. The remaining authors have not disclosed any potential conflicts of interests.

## Authors’ contributions

ZYP and JAK designed the study. ZYP, FHZ, and AC performed the animal experiments. JVB performed the flow cytometry, bacterial culture and counts, and *in vivo* labeling of PMNs. XYW performed the histology. MC performed biomarkers assays. JED performed biological imaging. FP performed the statistical analysis. GC and RP performed mathematical modeling. WF optimized and fabricated the blood-purification devices. AMK helped with imaging analysis and manuscript preparation. ME and KS contributed to the study conception and design, as well as analysis and interpretation of data. ZYP and JAK wrote the paper. All authors read and approved the final manuscript.
